# Circumstances and outcomes of falls among high risk community-dwelling older adults

**DOI:** 10.1186/2197-1714-1-5

**Published:** 2014-03-20

**Authors:** Judy A Stevens, Jane E Mahoney, Heidi Ehrenreich

**Affiliations:** 1 4770 Buford Highway NE, Mailstop F-62, Atlanta, GA 30341 USA; 2 Department of Medicine, University of Wisconsin School of Medicine and Public Health, Madison, WI 53705 USA

**Keywords:** Circumstances, Elderly, Falls, Fracture, Injury

## Abstract

**Background:**

For older adults, falls threaten their health, independence, and quality of life. Knowing the circumstances surrounding falls is essential for understanding how behavioral and environmental factors interact in fall events. It is also important for developing and implementing interventions that are effective and acceptable to older adults. This study investigated the circumstances and injury outcomes of falls among community-dwelling older adults at high risk for falls.

**Methods:**

In this secondary analysis, we examined the circumstances and outcomes of falls experienced by 328 participants in the Dane County (Wisconsin) Safety Assessment for Elders (SAFE) Research Study. SAFE was a randomized controlled trial of a community-based multifactorial falls intervention for older adults at high risk for falls, conducted from October 2002 to December 2007. Participants were community-dwelling adults aged ≥65 years who reported at least one fall during the year after study enrollment. Falls were collected prospectively using monthly calendars. Everyone who reported a fall was contacted by telephone to determine the circumstances surrounding the event. Injury outcomes were defined as none, mild (injury reported but no treatment sought), moderate (treatment for any injury except head injury or fracture), and severe (treatment for head injury or fracture).

**Results:**

Data were available for 1,172 falls. A generalized linear mixed model analysis showed that being aged ≥85 (OR = 2.1, 95% confidence interval [CI] = 1.2–3.9), female (OR = 2.1, 95% CI = 1.3–3.4), falling backward and landing flat (OR = 5.6, 95% CI = 2.9–10.5), sideways (OR = 4.6, 95% CI = 2.6–8.0) and forward (OR = 3.3, 95% CI = 2.0–5.7) were significantly associated with the likelihood of injury. Of 783 falls inside the home, falls in the bathroom were more than twice as likely to result in an injury compared to falls in the living room (OR = 2.4, 95% CI = 1.2–4.9).

**Conclusions:**

Most falls among these high risk older adults occurred inside the home. The likelihood of injury in the bathroom supports the need for safety modifications such as grab bars, and may indicate a need for assistance with bathing. These findings will help clinicians tailor fall prevention for their patients and have practical implications for retirement and assisted living communities and community-based fall prevention programs.

**Electronic supplementary material:**

The online version of this article (doi:10.1186/2197-1714-1-5) contains supplementary material, which is available to authorized users.

## Background

For older adults, falls and associated injuries threaten their health, independence and quality of life. More than a third of people aged 65 and older living independently fall each year (Tromp et al. 2001) and falls are the leading cause of injury-related deaths and hospital emergency department visits (CDC 2013).

While numerous fall risk factors have been identified (Rubenstein and Josephson 2002), more limited information is available about the detailed circumstances surrounding falls among community-dwelling older adults. Several studies have used retrospective survey data to analyze falls that occurred in the previous year (Morris et al. 2004Milat et al. 2011). Other studies have used prospective data but limited their focus to falls treated in emergency departments (Bleijlevens et al. 2010), falls among older women (Nachreiner et al. 2007), or falls that resulted in fracture (Luukinen et al. 2000) or hip fracture (Norton et al. 1997Allander et al. 1998).

This study used prospective, self-reported information about the circumstanced of falls among a group of high risk older adults and examined the relationships between location, activity, direction of the fall and subsequent injury.

## Methods

### The SAFE Study

This secondary analysis examined the circumstances and outcomes of falls among participants of the previously described Dane County (Wisconsin) Safety Assessment for Elders (SAFE) Research Study (Kiehn et al. 2009). This was a randomized controlled trial (RCT) of a community-based multifactorial falls intervention conducted from October 2002 to December 2007. Eligible participants were aged 65 and older, lived independently, and were at high risk of falls (defined as a person who, in the year before the SAFE study, had either experienced one fall with injury, two falls without injury, or one fall without injury and had balance problems). The University of Wisconsin Medical School human subjects committee approved the study. Informed consent was obtained before enrollment. People who were unable to give informed consent and who had no caregiver in their home to give consent for them were excluded.

Five hundred people were enrolled in the SAFE Study over a two year period and randomized to either treatment or control. The treatment group received an intervention that consisted of a multifactorial in-home falls assessment with recommendations, referrals for further care, and monthly telephone follow-up for one year. The intervention was provided by a physical therapist who had received two days of training regarding fall risk factors and interventions including those related to medications, low vision, and home and environmental changes. The control group received home safety education booklets and usual care. Participants were followed monthly for one year and 465 participants (93%) completed the one-year study.

Before randomization, a nurse went to each participant’s home and, after obtaining informed consent, collected baseline demographic and clinical data. Demographic information included age, gender, race, education, annual income, and living arrangement (live alone, with spouse, with family, or other). Baseline clinical data included cognition using the Short Portable Mental Status Questionnaire (SPMSQ) (Pfeiffer 1975), activities of daily living (ADL) using the Barthel Index (Mahoney and Barthel 1965), and balance confidence using the Activities-Specific Balance Confidence (ABC) Scale (Powell and Myers 1995).

Falls were the primary outcome measure and were assessed prospectively for one year. An unintentional fall was defined for participants as, “An event which results in a person coming to rest inadvertently on the ground or other lower level” (Gibson et al. 1987). Participants recorded all falls on a calendar (Tinetti et al. 1988) that they mailed to the study coordinator each month. In addition, participants kept monthly diaries to record falls circumstances and outcomes. All participants who indicated a fall on their calendar were contacted by telephone within one month and asked a series of questions about the circumstances and outcome of the fall. If a person was injured, he or she was asked specific questions about the type of injury and if he or she had sought medical care. So that researchers could better understand the circumstances, participants were asked to describe what happened at the time they fell, where they fell, their activity right before they fell, and in what direction they fell.

Falls that resulted from a violent blow, loss of consciousness, sudden onset of paralysis as in a stroke, or an epileptic seizure were excluded from the final data set (Gibson et al. 1987). Also excluded were falls that occurred in a hospital, nursing home or community-based residential facility.

### Falls circumstances analysis

We reviewed the narrative descriptions of the circumstances for each fall and created categorical variables that identified the general location (i.e., home, outdoors, in a public building), specific place within the general location (e.g., living room, bedroom, sidewalk), activity at the time of the fall (e.g., walking, standing up), direction of the fall (i.e., forward, sideways, backward to sitting, backward and landing flat, straight down), and the attributed cause as reported by the participant (e.g., lost balance, tripped.) If information about any of these variables was not available in the narrative, it was coded as “unspecified”.

All fall outcomes were based on self-report. We categorized a fall outcome as *none* if the participant reported no subsequent injury; “mild” if the person reported being injured but did not seek medical care; “moderate” if the person sought medical care for an injury other than a head injury or fracture; and “severe” if the person sought medical care for a self-reported head injury or fracture. We defined an injurious fall as one that resulted in any injury.

Data were analyzed using SAS (version 9.3). Chi-square statistics were used to test differences in categorical variables. We used a generalized linear mixed model that treated injury severity as a nominal three-level variable, (i.e., no injury, mild injury, and moderate or severe injury) to determine the odds ratios (OR) for circumstances associated with sustaining an injurious fall. The model took into account correlations between the falls of repeat fallers. The full model included age, gender, number of days in the study (excluding days spent in the hospital, nursing home, or community-based residential facility) (Tinetti et al. 1988) and the falls circumstances variables. The latter included the location of the fall, activity at the time of the fall, direction of fall, and attributed cause. Statistical significance was set at p < .05.

## Results

Of the 465 SAFE study participants, 328 (70.5%) reported at least one fall during the one-year follow-up period (122 fell once, 69 fell twice, 49 fell three times, and 88 fell four or more times); they provided information about the circumstances of 1,172 falls.

The baseline characteristics of the 328 fallers are shown in Table 1. About half (48.2%) were between 75 and 84 years of age, almost three-quarters (72.3%) were female and 59.5 percent lived alone. The sample was 97.2 percent white, which reflected the catchment area population. Overall, the group had little cognitive impairment, as indicated by an average score on the SPMSQ of 0.8 ± 1.8 on a scale of 0–10 (maximum impairment = 10) (Pfeiffer 1975). The participants had minor limitations in their activities of daily living (ADL), with an average Barthel Activities Score of 88 ± 18 on a scale of 1–100 (maximum functional score = 100) (Mahoney and Barthel 1965). However, they had only a moderate level of confidence in being able to maintain their balance during activities, as shown by an average score on the modified Activities–specific Balance Confidence (ABC) scale of 6.0 ± 2.1 on a scale of 1–10 (maximum confidence score = 10) (Powell and Myers 1995).

**Table 1:** **Baseline characteristics of 328 fallers aged 65 and older**

Demographic	% ^*^
Age	
65–74	30.2
75–84	48.2
85+	21.6
Gender	
Female	72.3
Race	
White	97.2
African–American	1.0
Other	1.8
Annual income	
0–9,999	9.5
10,000–24,999	35.7
25,000–49,999	24.7
50,000+	18.0
DK/Refused	12.2
Living arrangement	
Alone	59.5
With spouse	30.8
With family	7.0
Other	2.7
Years of school completed (mean ± SD)	14.3 ± 4.0
**Clinical**	Mean ± SD
Short Portable Mental Status Questionnaire (SPMSQ). (Maximum impairment score = 10) (Pfeiffer 1975)	0.8 ± 1.8
Barthel Activities of Daily Living (ADL) score. (Maximum functional score = 100) (Mahoney and Barthel 1965)	88 ± 18
Modified Activities-specific Balance Confidence (ABC) score. (Maximum confidence score = 10) (Powell and Myers 1995)	6.0 ± 2.1

^*^Rounding may result in totals slightly over or under 100%.

Injury severity differed by SAFE participant status. Intervention participants sustained 44.8% of all falls (525/1172) and 56.2% of the moderate or severe injuries (50/89) while control participants sustained 55.2% of all falls and 43.8% (39/89) of moderate or severe injuries. Although these differences were statistically significant (chi square p = .01), there was no protective effect of having been in the intervention group.

The general location of the fall, (e.g., inside their or another person’s home, outdoors, in a public building) did not differ by gender (chi square p = 0.15) or by their participant status in the SAFE Study (intervention or control) (chi square p = 0.14) (data not shown). Therefore, the falls were pooled in subsequent analyses. However, the location of the fall did differ by age group. People aged 85 and older were significantly more likely to fall inside their home than were younger people (age 65–74 [62.5%]; 75–84, [67.7%]; ≥85 [73.9%]) (chi square p = .03).

Table 2 shows the outcomes of 1,172 falls by a number of demographic and fall characteristics. Falls occurred most often among people aged 75 to 84 years. The proportions of mild compared to moderate or severe injuries were similar for persons 75 to 84 (47.8% mild vs. 43.8% moderate or severe). However, for people 85 and older, the greatest proportion of injuries were moderate or severe (18.9% mild vs. 34.8% moderate or severe). Women sustained 56.3 percent of the reported falls but experienced 75.3 percent of the falls that resulted in moderate or severe fall injuries.

**Table 2:** **Injury severity of 1,172 falls by age, gender, location, attributed cause, activity and direction of the fall**

	Injury severity*							
	None		Mild		Moderate or severe		Total	
	N = 782	(%)**	N = 301	(%)	N = 89	(%)	N = 1172	(%)
**Age group**								
65–74	316	(40.4)	100	(33.2)	19	(21.4)	435	(37.1)
75–84	333	(42.6)	144	(47.8)	38	(43.8)	515	(43.9)
≥85	133	(17.0)	57	(18.9)	31	(34.8)	221	(18.9)
**Gender**								
Men	386	(49.4)	104	(34.6)	22	(24.7)	512	(43.7)
Women	396	(50.6)	197	(65.5)	67	(75.3)	660	(56.3)
**Location**								
Outside	197	(25.2)	82	(27.2)	30	(33.7)	309	(26.4)
In a public building	57	(7.3)	21	(7.0)	2	(2.3)	80	(6.8)
Inside the home	528	(67.5)	198	(65.8)	57	(64.0)	783	(66.8)
**Activity**								
Walking	214	(27.4)	80	(26.6)	27	(30.3)	321	(27.4)
Standing up	100	(12.8)	25	(8.3)	8	(9.0)	133	(11.3)
Stepping up or down (stairs/step/curb/ladder/stepstool)	55	(7.0)	38	12.6	7	(7.9)	100	(8.5)
Reaching or leaning	61	(7.8)	12	(4.0)	4	(4.5)	77	(6.6)
Turning or changing direction	48	(6.1)	20	(6.6)	6	(6.7)	74	(6.3)
Bending or pushing	37	(4.7)	9	(3.0)	3	(3.4)	49	(4.2)
Other specified	212	(27.2)	102	(33.9)	26	(29.2)	340	(29.0)
Not specified	55	(7.0)	15	(5.0)	8	(9.0)	78	(6.7)
**Direction of the fall**								
Forward	327	(41.8)	133	(44.2)	36	(40.5)	496	(42.3)
Sideways	134	(17.1)	86	(28.6)	20	(22.5)	240	(20.5)
Backward to sitting	174	(22.3)	27	(9.0)	5	(5.6)	206	(17.6)
Backward and landing flat	71	(9.1)	39	(13.0)	12	(13.5)	122	(10.4)
Straight down	17	(2.2)	2	(0.7)	2	(2.3)	21	(1.8)
Other specified	12	(1.5)	1	(0.3)	0	0	13	(1.1)
Not specified	47	(6.0)	13	(4.3)	14	(15.7)	74	(6.3)
**Attributed cause**								
Lost balance, unsteady or wobbly	259	(33.1)	87	(28.9)	23	(25.8)	369	(31.5)
Trip, caught foot, clumsy or tangled feet	208	(26.6)	100	(33.2)	26	(29.2)	334	(28.5)
Slip	69	(8.8)	23	(7.6)	8	(9.0)	100	(8.5)
Legs or hip gave out, rubbery legs or leg weakness	64	(8.2)	17	(5.7)	4	(4.5)	85	(7.3)
Other specified	96	(12.3)	45	(15.0)	13	(14.6)	154	(13.1)
Not specified	86	(11.0)	29	(9.6)	15	(16.9)	130	(11.1)

*Mild: Reported injury but did not seek treatment; Moderate: Sought medical care for injuries excluding head injuries and fractures; Severe: Sought medical care for head injuries and fractures.

**Rounding may result in totals slightly over or under 100%.

Of 389 falls that occurred outside the home, 309 were outdoors and 80 were in public buildings. Of falls that occurred outdoors, 31.7 percent occurred in areas characterized as a garden, lawn, or woods, 19.9 percent happened on outdoor stairs or steps, and 18.8 percent on sidewalks or driveways (data not shown). Of the 80 falls that occurred inside public buildings, 15.6 percent happened in stores, 11.7 percent in recreational settings, and 10.4 percent in hotels or motels (data not shown). Falls that caused moderate or severe injuries occurred most often while people were walking (30.3%) or when standing up (9.0%). However, 29.0 percent of all falls, regardless of outcome, happened while people were engaged in diverse “other specified” activities, (e.g., cleaning, opening or closing doors, bathing, getting into or out of a car). The direction of the fall appeared to be associated with injury outcomes. The largest proportion of moderate or severe injuries occurred when falling forward (40.5%) and sideways (22.5 %) (Table 2). Falling backward to sitting or straight down resulted in the smallest proportion of moderate or severe injuries. People most often attributed their falls to either losing their balance (31.5 %) or tripping (i.e., catching their foot on something) (28.5 %), and only 8.5 percent of falls were attributed to slipping (i.e., sliding or losing their footing). However, no cause was given for 11.1 percent of falls.

Two thirds of falls (783 or 66.8 %) occurred inside the home. For each injury outcome, (i.e., no injury, mild injury, moderate or severe injury), we examined the distribution of falls within the main rooms of the home. After excluding unspecified locations, we found no statistical difference in the distributions for mild vs. moderate or severe injuries, so these injury categories were combined.

**Figure 1:**
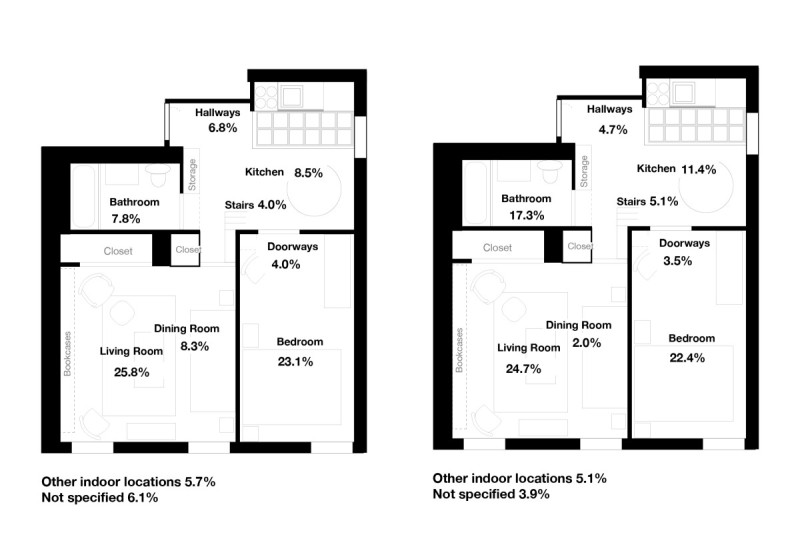
**Distribution of falls with and without injuries that occurred inside the home.**

In Figure 1, a. illustrates the distribution within the home of 528 falls with no injury and b. shows the distribution of 255 falls with any (mild, moderate or severe) injury. Regardless of whether an injury occurred, the largest proportion of falls happened in the living room and bedroom. However, 41 of 528 (7.8%) of falls with no injury and 44 of 255 (17.3%) of falls with any injury occurred in the bathroom, a statistically significant difference (chi square p < .001).

To assess the characteristics associated with sustaining an injurious fall, we used a generalized linear mixed model that included age-group, gender, location, number of days in the study, activity, attributed cause, and direction of fall (Table 3). Age-group, gender, and direction of fall were statistically significant. People aged 85 and older were twice as likely as people aged 65 to 74 to sustain a fall injury (p = .01) and women were twice as likely as men to be injured (p = .001).

**Table 3:** **Generalized linear mixed model** ^*^ **of characteristics significantly associated with injurious** ^**^ **falls among people aged 65 years and older**

Falls characteristic	Odds ratio	95% Confidence interval
**Age group**		
65–74	Ref	–––
75–84	1.7	1.0–2.8
≥85	2.2	1.2–3.9
**Gender**		
Male	Ref	–––
Female	2.1	1.3–3.4
**Direction of the fall**		
Backward to sitting	Ref	–––
Backward and landing flat	5.6	2.9–10.5
Sideways	4.6	2.6–8.0
Forward	3.3	2.0–5.7
Straight down	1.7	0.4–6.9
Other specified	0.6	0.1–6.1
Not specified	5.1	2.4–10.9

^*^Model included age-group, gender, location, number of days in the study, activity, attributed cause, and direction of the fall.

^**^Injury severity (none, mild, moderate/severe) treated as a nominal variable.

The likelihood of an injury was strongly associated with the direction of the fall. For example, falling backward and landing flat was about five and a half times more likely to result in an injury compared to falling backward into a sitting position (i.e., “Her leg gave out and she fell and landed on her bottom”). Similarly, an injury was about four and a half times more likely to result from falling sideways and three times more likely from falling forward. In addition, falls without a specified direction were significantly more likely to result in an injury, compared to falling backward into a sitting position.

When we limited the model to the 783 falls that occurred inside the home and included specific locations (i.e., living room, bedroom, kitchen, bathroom, all other locations combined), we saw similar results (Table 4). Older age, female gender, and direction of the fall again were significantly associated with the likelihood of sustaining an injury. In addition, compared to falls in the living room, falls in the bathroom were almost two and a half times more likely to result in an injury (OR = 2.4, 95% CI = 1.2-4.9).

**Table 4:** Generalized linear mixed model* of characteristics significantly associated with injurious** falls in the home among people aged 65 years and older

Falls characteristic	Odds ratio	95% Confidence interval
**Age group**		
65–74	Ref	–––
75–84	2.1	1.1–3.8
≥85	2.1	1.0–4.3
**Gender**		
Male	Ref	–––
Female	2.0	1.1–3.5
**Location**		
Living room	Ref	–––
Bedroom	1.1	0.6–1.9
Kitchen	1.4	0.7–2.7
Bathroom	2.4	1.2–4.9
Other specified	0.7	0.4–1.3
**Direction of the fall**		
Backward to sitting	Ref	–––
Backward and landing flat	4.8	2.2–10.5
Sideways	3.0	1.5–5.9
Forward	2.2	1.2–4.3
Straight down	1.6	0.3–7.9
Other specified	0.4	0.3–6.9
Not specified	5.0	2.0–12.6

^*^Model included age-group, gender, room, number of days in the study, activity, attributed cause, and direction of the fall.

^**^Injury severity (none, mild, moderate/severe) treated as a nominal variable.

## Discussion

This study investigated 1,172 falls sustained by 328 community-dwelling older adults at high risk of falls who had fallen during the course of a year, and examined the circumstances that resulted in no, mild, and moderate or severe injuries. Using a generalized linear mixed model, we identified three significant variables: age-group, gender and direction of the fall. Being aged 85 and older or being female doubled the likelihood that a fall would result in an injury. Compared to falling backward into a sitting position, injuries were most likely to result from falling backward and landing flat, falling sideways and, to a somewhat lesser extent, falling forward. When we looked only at falls that occurred inside the home, falls in the bathroom were more than twice as likely to result in an injury, compared to falls in the living room.

It is well documented that women are more likely than men to sustain nonfatal fall injuries (CDC 2013). In 2011, after adjusting for age, the fall injury rate for women treated in U.S. emergency departments was 46 percent higher than for men (CDC 2013). In a population-based study of gender differences, Stevens and Sogolow (2005) found that nonfatal fall injury rates for women were 40 to 60 percent higher than for men of comparable age. For both men and women, fall injury rates increased sharply with age with the greatest increase occurring after age 80 (CDC 2013).

In this study, about 26 percent of falls occurred outdoors compared to as much as 50 percent reported in other studies (Kelsey et al. 2010Bergland et al. 2003). Research has demonstrated that healthy active older adults are more likely to fall outdoors (Mänty et al. 2009Bleijlevens et al. 2010Kelsey et al. 2012). Our participants were at high risk for falls, had some limitations in ADLs as well as limited self-confidence about falling, so we would expect to see a lower percentage of falls outdoors.

We identified locations within the home where fall injuries occurred, an approach recommended by Runyan et al. (2005) to improve data collection of home injuries. We found that 17 percent of injurious falls, compared to eight percent of non-injurious falls, occurred in bathrooms. The likelihood of sustaining a fall injury in the bathroom was almost two and a half times that of experiencing a fall injury in the living room. It is reasonable that falling in a small room with porcelain surfaces, metal fixtures, and hard floors would be more likely to result in an injury than falling in a larger area with upholstered furniture and/or carpeted surfaces.

In a cross-sectional study, Bleijlevens et al. (2010) assessed 333 older adults treated in emergency departments after a fall and found that about ten percent of fall-related fractures were associated with going to, coming from, or being in the bathroom. These fallers also were the most inactive. Similarly, an analysis of nonfatal bathroom injuries treated in U.S. emergency departments found that about 81 percent of these injuries were caused by falls. The highest injury rate was among people aged 65 and older, and injuries occurred most frequently when people were in or getting out of the tub or shower, and when they were standing up, sitting down, or using the toilet (Stevens et al. 2011).

This is the first study to show that, among falls inside the home, those in the bathroom were most likely to result in an injury. These findings support the need for improving safety in the bathroom. This may include, 1) getting assistance from another person for bathing; 2) adopting safer methods when carrying out activities in the bathroom (e.g., wearing shoes with non-slip soles, storing toiletries on easy-to-reach shelves, using an assistive device safely), and 3) using and/or installing safety equipment (e.g., non-skid tub or shower mats, grab bars both inside and outside the tub or shower and around the toilet, and raised toilet seats).

An effective intervention, shown in a number of RCTs to reduce falls in the home, to is to have an occupational therapist (OT) conduct an in-home safety assessment (Stevens 2010). This may be covered by Medicare if the person previously has been injured in a fall. An OT can evaluate a person’s ability to perform daily activities in their home, teach the individual how to accomplish these activities more safely, and/or make suggestions for home modifications to reduce potential fall hazards. Such behavioral and environmental changes can prevent falls and subsequent injuries. In addition, depending on a person’s level of risk, fall prevention interventions that have been shown in RCTs to effectively reduce falls include individualized exercises prescribed by a physical therapist; home-based progressive exercise programs, such as the Otago Exercise Program; and community exercise programs that improve balance and lower body strength, such as Tai Chi. (Rose 2008Sherrington et al. 2008Stevens 2010)

We found a strong association between the direction of the fall and the likelihood of injury. Others have not seen this relationship (Demura et al. 2012), but these researchers did not distinguish falls backward into a seated position (low risk for injury) from those backward and landing flat (higher risk for injury). In our study, falling backward and landing flat was about five and a half times as likely to result in an injury, and falling sideways was four times as likely, compared to falling backward and landing in a sitting position. Falling backward and landing flat may result in head injury while falling sideways can cause hip fracture (Hayes et al. 1993). These severe injuries often result in long-term functional impairment, nursing home admission and increased mortality (Magaziner et al. 1990Thompson et al. 2006Penrod et al. 2008). Of 1,172 reported falls in the current study, only 29 (2.5%) caused fractures or head injuries. Therefore, we were not able to assess the relationship between these specific types of serious injuries and the direction of the fall.

One-third of falls in this study were attributed to tripping or slipping. Promising falls interventions include techniques for teaching individuals in laboratory settings how to regain their balance (Pai and Bhatt 2007Mansfield et al. 2010Wang et al. 2011), although these may not be practical on a population level. Recent work with healthy older adults showed that under laboratory conditions, training that used surface perturbations to simulate slipping and induce backwards falls improved both proactive (pre-slip) and reactive (post-slip) balance strategies. The result was fewer backward falls (Mansfield et al. 2010Wang et al. 2011).

Grabiner et al. (2012) explored whether task-specific training could reduce trip-related falls among 52 healthy middle-aged and older women. Using a treadmill to simulate tripping, the 22 women who had received training had significantly fewer falls (4.5%) than the 30 control women (26.6%). However, it is not known if laboratory training would benefit less healthy older adults or if it would translate into fewer falls from unexpected trips and slips in real life settings. Further research is needed to determine practical interventions that can decrease falls from tripping and slipping.

Using a multivariate model, we did not find an association between self-reported activity and likelihood of a fall injury. Falls occurred most often when a person was walking, a finding that has been reported previously (Berg et al. 1997Nachreiner et al. 2007Milat et al. 2011). However, it is difficult to compare studies because the activities described often depend on the population (e.g., healthy vs. less-healthy older adults) and the location (e.g., outdoors vs. indoors) (Kelsey et al. 2010Kelsey et al. 2012). We categorized each activity based only on what was in the person’s recorded narrative. We were unable to classify many of the “other specified” activities because it would have required making assumptions about the underlying activity, (e.g., assuming that “cleaning” was essentially the same activity as “reaching”.) Some participants described a sequence of events in which one activity lead to subsequent events that culminated in a fall, which made it difficult to establish the activity at the time of the fall. Similar issues made it difficult to classify the attributed causes of falls, (e.g., trip, slip, lost balance, legs gave out, etc.) In addition, the causes of almost one-third of falls were nonspecific and attributed only to a loss of balance.

This study has several limitations. There was inadequate detail available about the circumstances of some falls, although there had been a concerted effort to collect these data. Although the study excluded falls due to syncope, some such falls may have been included if the participant did not remember experiencing a loss of consciousness (Shaw and Kenny 1997). The direction of six percent of falls was unspecified and these were more likely to be injurious falls. For participants who were hospitalized following a fall injury and then admitted to a rehabilitation facility, there was often a delay in obtaining information about the fall. This may have resulted in poorer recall of the fall circumstances. Also, this study included people at high risk for falls and the results cannot be generalized to all community-dwelling older adults.

A major strength of this study is that data about falls were collected prospectively using monthly calendars, a method that is considered the gold standard (Ganz et al. 2005Hauer et al. 2006). Obtaining fall data retrospectively can result in underreporting. In one study, two-thirds of people who had sustained an injurious fall did not recall the injury when questioned six months later (Mackenzie et al. 2006). Our study used monthly calendars that were mailed each month to the study coordinator. All participants who reported a fall were contacted by telephone to ascertain the circumstances and extent of any injury. Telephone interviews for falls circumstances have demonstrated good agreement with face-to-face interviews (Mackintosh et al. 2009).

## Conclusions

This study of older adults at high risk for falls found that most falls occurred at home and that women and people aged 85 and older were most likely to be injured. There was a greater likelihood of injury from falling forward, sideways, and backward and landing flat, compared to falling backward to sitting. And finally, for falls inside the home, there was a significantly greater likelihood of sustaining an injury in the bathroom compared to the living room. These results support the need to promote safety modifications such as grab bars and may indicate a need for assistance with bathing. These findings will help clinicians tailor fall prevention for their patients and have practical implications for retirement and assisted living communities as well as for community-based fall prevention programs.

## Authors’ original submitted files for images

Below are the links to the authors’ original submitted files for images. Authors’ original file for figure 1
