# Supplementary figures and images for: Circumstances and outcomes of falls among high risk community-dwelling older adults
Source: Inj Epidemiol. 2014 Mar 20;1:5. doi: 10.1186/2197-1714-1-5 (PMC4700929; doi:10.1186/2197-1714-1-5)

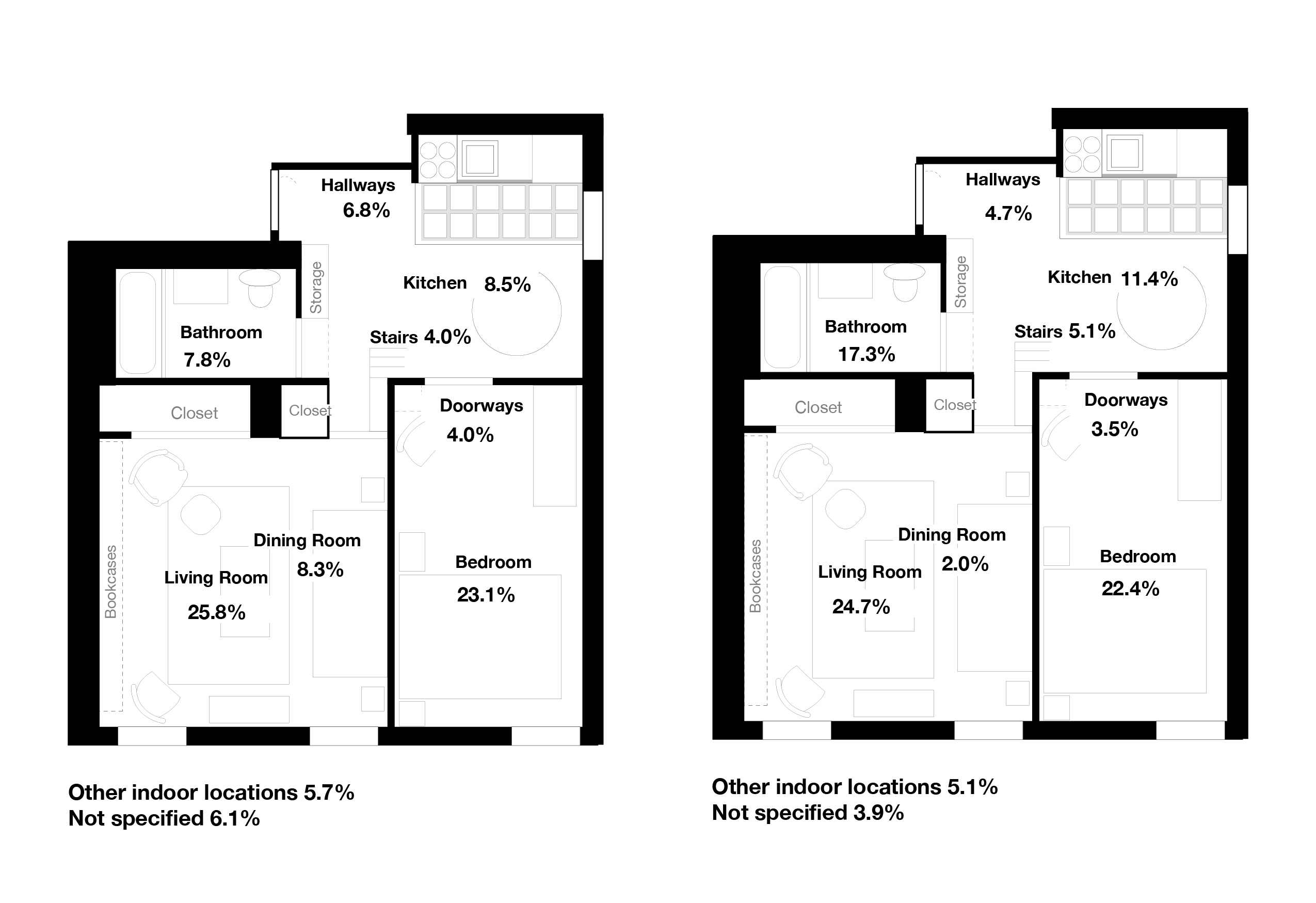

Supplement: Supplementary file 1 — Authors’ original file for figure 1 [file 40621_2013_5_MOESM1_ESM.jpeg]
